# Negative interaction effect of heat and drought stress at the warm end of species distribution

**DOI:** 10.1007/s00442-023-05497-5

**Published:** 2024-01-23

**Authors:** Judith R. Schepers, Jessica Heblack, Yvonne Willi

**Affiliations:** Department of Environmental Sciences, University of Basel, 4056 Basel, Switzerland

**Keywords:** Adaptation, Drought stress, Heat stress, Phenotypic selection, Warm range limit

## Abstract

Geographic range limits of species are often a reflection of their ecological niche limits. In many organisms, important niche limits that coincide with distribution limits are warm and warm-dry conditions. We investigated the effects of heat and drought, as they can occur at the warm end of distribution. In a greenhouse experiment, we raised North American *Arabidopsis lyrata* from the centre of its distribution as well as from low- and high-latitude limits under average and extreme conditions. We assessed plant growth and development, as well as leaf and root functional traits, and tested for a decline in performance and selection acting on growth, leaf, and root traits. Drought and heat, when applied alone, lowered plant performance, while combined stress caused synergistically negative effects. Plants from high latitudes did not survive under combined stress, whereas plants originating from central and low latitudes had low to moderate survival, indicating divergent adaptation. Traits positively associated with survival under drought, with or without heat, were delayed and slowed growth, though plastic responses in these traits were generally antagonistic to the direction of selection. In line, higher tolerance of stress in southern populations did not involve aspects of growth but rather a higher root-to-shoot ratio and thinner leaves. In conclusion, combined heat and drought, as can occur at southern range edges and presumably more so under global change, seriously impede the long-term persistence of *A. lyrata*, even though they impose selection and populations may adapt, though under likely interference by considerable maladaptive plasticity.

**Supplementary Information:**

The online version contains supplementary material available at 10.1007/s00442-023-05497-5.

## Introduction

Across the globe, temperatures have been increasing and precipitation has become more variable, with more droughts or extreme rain (IPCC 2023). In turn, warming has been linked to the retreat of some species from the warm limits of their distribution (Parmesan 2006; Cahill et al. 2014; Sánchez-Salguero et al. 2017; Rumpf et al. 2018). Causes of retreat can include the direct effect of abiotic stressors, biotic stressors, or interactions among them (Cahill et al. 2014; Paquette and Hargreaves 2021). Populations often evolve particular strategies to cope with one type of stressor over their evolutionary histories, which can interfere with strategies for coping with more extreme stress or other stressors (Fry 2003; Ågren and Schemske 2012; Santos del Blanco et al. 2013; Willi and Van Buskirk 2022). For example, it was shown that combined stressors, such as heat and drought, can act to amplify negative effects (Craufurd and Peacock 1993; Savin and Nicolas 1996; Dreesen et al. 2012; Zandalinas and Mittler 2022). Consequently, if we aim to understand why species fail to cope with extreme conditions at the warm end of species distributions, stressors need to be studied both individually and in combination (Suzuki et al. 2014).

Plants have evolved various ways of coping with heat, which have been studied in regards to the genes involved, the physiology, morphology, and development (Berry and Bjorkman 1980; Bita and Gerats 2013; Zhao et al. 2021; Sher et al. 2022; Yadav et al. 2022). In many species, a general strategy of coping with heat is leaf cooling through increased transpiration (Crawford et al. 2012; Deva et al. 2020; Sadok et al. 2021). Increased transpiration is achieved by a longer stomatal opening and higher stomatal conductance (Marchin et al. 2022). Such cooling requires a continuous supply of water, which is ensured, for example, by deep roots, an extensive and complex root system, or by a high root-to-shoot ratio (Parker 1949; Aston and Lawlor 1979; Natarajan and Kuehny 2008; Giri et al. 2017). Strategies affecting morphology are generally targeted at decreasing surface area to reduce the area of water loss by thick stems and leaves, short internode lengths, or smaller leaves (Vile et al. 2012; Stewart et al. 2016; Leigh et al. 2017). Coping with heat may also include a faster phenology, such as early flowering to escape the heat during critical life stages (e.g., in *Arabidopsis thaliana*, Balasubramanian et al. 2006; Taylor et al. 2019). Additionally, leaf pigments can play an important role during heat and high irradiation as paler leaves with less chlorophyll help maintain energy balance and lower the risk of overheating (Kume 2017; Genesio et al. 2020), while carotenoids can dissipate excess energy and thereby protect the chlorophyll apparatus (Kumar et al. 2020).

Plants have evolved also various strategies to cope with drought (Murtaza et al. 2016), which sometimes differ substantially from those of coping with heat (Zhang and Sonnewald 2017). Under drought conditions, an immediate reduction of water-loss is achieved by the closure of the stomata; this ensures that the leaf water potential does not drop to critical levels and that plant metabolic processes are maintained (Verslues and Juenger 2011; Tardieu 2013). In combination with increased water uptake from the soil, the plant can thus maintain the physiological water balance (Rodrigues et al. 2019). Increased water uptake during a short period of drought is achieved by a wider and deeper root system (Dinneny 2019). In addition to longer roots, smaller leaves are a common response of plants growing under drought conditions, leading to an increased root-to-shoot ratio and reduced leaf surface area per dry weight (lower specific leaf area, SLA) (Matsui and Singh 2003; Dovrat et al. 2019). Another adjustment to a dry climate is accelerated reproductive development (Franks et al. 2007). Further strategies related to escape include a shorter growth period, earlier germination, or dormancy during extreme events (Basu et al. 2016; Franks 2011; Verslues and Juenger 2011; Tardieu 2013; Balachowski et al. 2016).

Combined heat and drought may be particularly challenging for plants. Marchin et al. (2022) reported for broadleaf evergreens that stomata closure is of advantage during drought, as it can maintain a high water potential of the leaves, but it can lead to overheating of leaves under heat. Conflicting responses to heat and drought were also reported for *A. thaliana* (cv Columbia) and *Nicotiana tabacum* (Rizhsky et al. 2002, 2004). While plants responded to heat by increased photosynthesis and respiration, they responded to drought by reducing both processes. Under combined heat and drought, plants increased respiration but reduced photosynthesis, leading to senescence. Also in *A. thaliana*, high temperatures and the combination of heat and water deficiency accelerated reproductive development, while water deficiency alone delayed reproduction (Vile et al. 2012). The different responses to heat, drought and both stressors in combination confirm the need to investigate single and combined stressors to reveal the conflicts among strategies that impede their fitness benefits, particularly in the face of global warming.

The response to climatic stress often depends on the climate history of populations and can therefore vary greatly within species (Lexer et al. 2003). Indeed, local climate has been linked with adaptive differences among populations in several studies (e.g., Richardson et al. 2014; Estarague et al. 2022; Sánchez-Castro et al. 2022). Adaptive differences may be expressed under stress, but also when plants grow under ideal climatic growth conditions. In the canopy species *Corymbia calophylla* and in *A. thaliana*, plants originating from hot and/or dry areas differed in trait expression even under benign conditions; they had lower SLA, higher leaf dry matter content (LDMC), or smaller leaf area (May et al. 2017; Ahrens et al. 2020). Another aspect of climate adaptation is that within species or closely related species, there may be differences in how it is achieved. For example European *A. lyrata* subsp. *petraea* of southern range edges was shown to flower earlier and have a higher flowering propensity (Riihimäki and Savolainen 2004), while in North American *A. lyrata* subsp. *lyrata*, plants from northern latitudes have faster reproductive development (Paccard et al. 2014).

**Fig. 1:**
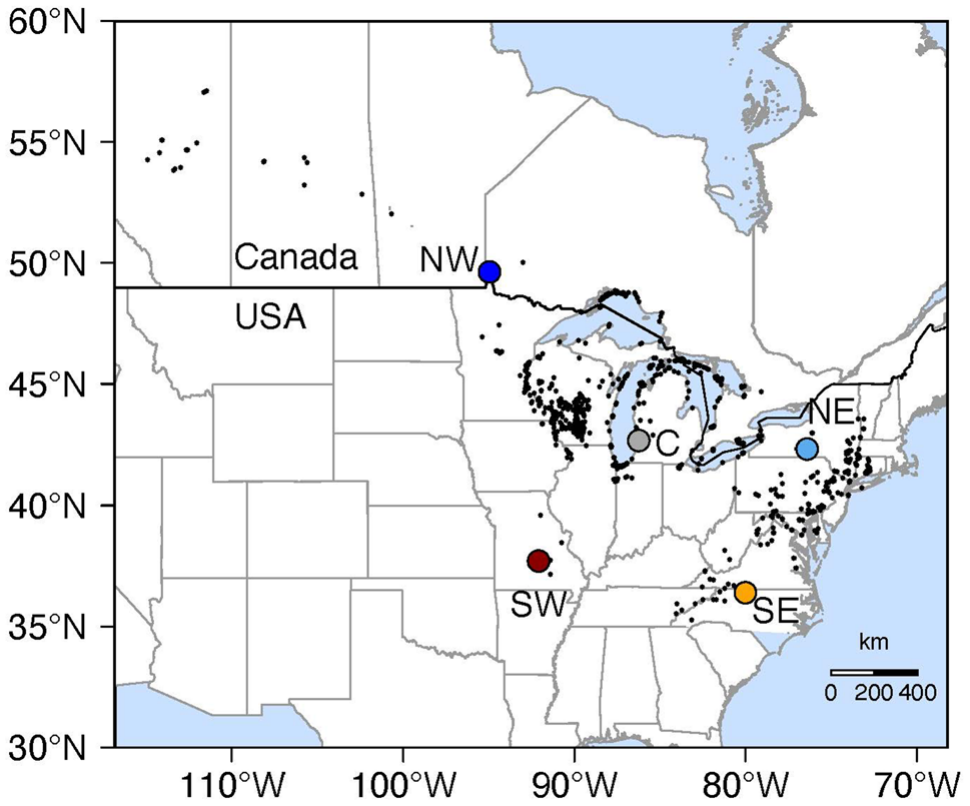
Range of *Arabidopsis lyrata* in North America. The black dots indicate species occurrences reported since 1960 of a thinned dataset. Coloured dots show the locations of the populations used in this study: one from the centre of the range (C), and the others from the range edges, from the north-east (NE), north-west (NW) south-east (SE) and south-west (SW)

The aim of this study was to test whether heat, drought and combined stress had similar effects on growth, leaf and root functional traits, whether populations responded differently depending on their climate of origin, and whether plastic changes were in the direction favoured by selection. The study organism was the North American *Arabidopsis lyrata* spp. *lyrata* (hereafter *A. lyrata*). Environmental niche modelling had revealed that the range limits of *A. lyrata* in the south and the north were associated with climate niche limits, with minimum temperature in early spring being the most niche- and range-limiting factor (Lee-Yaw et al. 2018). But with climate change, temperature and precipitation have changed across the distribution area of *A. lyrata*, resulting in reduced environmental suitability at the southern distribution limit (Online Resource 1 Fig. S1, Online Resource 2 Table S1). We analysed the stress responses of five populations, one from the range centre and two each from the warm and cold ends of the species’ distribution (Figs. 1, Online Resource 1 S1, Online Resource 2 Tables S1, S2). Plants were grown in the greenhouse under four distinct temperature and watering conditions, based on average or higher temperature and average or lower precipitation as they occur at the low-latitude range edge during the growing season (Online Resource 2 Table S1). We addressed the following questions: (1) Do heat, drought, and heat-drought differ in how they affect growth, leaf and root functional traits, and do responses vary among populations and seed families within populations? (2) What is the difference in trait expression in populations from the southern edge as compared to central and northern populations? Are trait differences between these groups of populations the same as the plastic changes? And (3) how does selection act on traits? Does selection in the different environments align with plastic changes?

## Materials and methods

### Plant material

*Arabidopsis lyrata* subsp. *lyrata* is native to the eastern and mid-western United States and south-eastern Canada, and it is locally restricted to substrates with little water-holding capacity, sand and rocky outcrops (Koch et al. 2001; Al-Shehbaz and O’Kane 2002; Schmickl et al. 2010). Seeds were collected from five *A. lyrata* populations (Fig. 1): a genetically highly diverse one from the centre of the range (**C**) (Wos and Willi 2018), and four from the edges, in the north-east (**NE**), north-west (**NW**), south-east (**SE**), and south-west (**SW**, details in Table S2). Collections were performed between 2007 and 2014, and seeds of field-collected plants were propagated together during one generation in the greenhouse by performing crosses within unique pairs of plants of the same population. For this experiment we considered three pairs of plants for range-edge populations and 120 pairs for the central population; the latter population was used for selection analysis and therefore included many more plants.

### Climate data

Climate data at the sites of the five populations were obtained from WorldClim v1.4 (Hijmans et al. 2005), v2.1 (Fick and Hijmans 2017) and CRU-TS 4.06 (Harris et al. 2020) downscaled with WorldClim v2.1. We downloaded monthly average temperature (T_mean_), maximum temperature (T_max_), precipitation (P) and precipitation during the driest month (P_min_, Bio14) for the periods of 1960–1990 and 1970–2000. For 2000–2018, we used the monthly minimum temperature (T_min_), maximum temperature (T_max_), and precipitation (P), and calculated monthly average temperature (T_mean_) and precipitation of the driest month (P_min_). For T_mean_, T_max_ and P of the three time periods, we calculated averages for the months of April to June and June to August (using the dplyr and raster packages; Hijmans 2022; Wickham et al. 2022). For the first two periods, the resolution was 30 s, for 2000–2018, the resolution was 2.5 min. Plots and all statistics were done with R (R-Core-Team 2021). Raster plots (Figs. 1, Online Resource 1 S1) were produced with the R packages sp and sf (Pebesma and Bivand 2005; Pebesma 2018).

### Experimental design

Offspring plants were grown under four climatic conditions in a two-by-two factorial design, with average or high temperature, and average or low precipitation as occurs at the two warm-end populations (**SE** and **SW**) (Online Resource 1 Fig. S1). We assumed that plants would germinate during fall or early spring and grow and develop thereafter. To imitate average conditions, values close to mean temperature and precipitation for April to June were chosen (data in Online Resource 2 Table S1). For the heat treatment, temperature was set close to the mean of June to August. For the drought treatment, precipitation of the driest month for the two sites was taken.

For each treatment combination, five blocks were set up, each with one replicate seed per cross (edge populations were only represented in three blocks). Seeds were placed into 54-multipot trays within a block, filled with a sand-peat mixture of 2:1. Only every second pot of a tray was used to prevent plants from growing into each other and to facilitate image analysis. Seeds were stratified for 12 days at 4 °C in climate chambers at 70% humidity (ClimeCab 1400, KÄLTE 3000 AG, Landquart, Switzerland) and then transferred to four greenhouse chambers (temperature of 18 °C). During stratification and germination, plants were covered with mesh nets to maintain high humidity. To ensure a gradual change between stratification and experimental conditions, day length was increased from 8 h to 1 h every 3–4 days until the day length was 16 h, with a light intensity of 200 μM s^−1^ m^−2^. During the transition phase, day temperature was 20 °C and night temperature was 18 °C, and plants were watered daily by spraying from above. After 7 days, when approximately 75% of the plants had germinated, the mesh nets were removed. After an additional 14 days, when about 80% of germinated plants were at the four-leaf stage, stress treatments began.

Two of the four greenhouse chambers (University of Basel greenhouse) were set to have cold temperatures, and two to have warm temperatures. Each chamber of a particular temperature regime contained either two or three spatial blocks of multi-pot trays with plants of both watering treatments. Based on climate data from the two southern sites (Online Resource 2 Table S1), we set the low-temperature regime to an average of 20.6 °C: 22 °C during the day, a one-hour heat peak of 25 °C at noon, and night temperature at 18 °C for 8 h. The high-temperature regime had an average of 25.2 °C: 27 °C during the day, a heat peak of 30 °C at noon, and a night temperature of 23 °C. The high precipitation/watering regime was initially 8.4 ml of water every second day, corresponding to 100 mm m^−2^ of monthly precipitation. The low-precipitation treatment was 5 ml of water every second day corresponding to 65 mm m^−2^ of monthly precipitation. Due to sudden early dieback in the dry treatment because the soil in the small pots dried out quickly, watering was increased by 20%, to 10 ml and 6 ml in the high and the low precipitation regimes, respectively; in nature, soil bodies where *A. lyrata* grows are typically deeper and less likely to dry out as rapidly. In all chambers, air humidity was set to 70%. Trays were randomized twice per week (within blocks, and block position in the paired chambers), and fertilizer was given every 4 weeks (0.2% Wuxal universal fertilizer, Westland Schweiz GmbH, Dielsdorf, Switzerland). Additionally, after 14 weeks, an insecticide (1.5% Kendo gold, Westland Schweiz GmbH) was applied once a week to protect the plants from insect infestations.

### Trait assessment

Performance. After stratification, every day for 2 weeks we recorded the day of germination, when the cotyledons became visible. Afterwards, pots were examined every second to third day for further germination, death (all leaves brown and dry), bolting (visible flowering stem), flowering (first flower), revival of plants (green leaves), and infestation. This approach resulted in data on days to germination, survival, longevity (days until death or harvest), and flowering propensity.

Growth traits. We monitored the growth of rosettes by taking images twice a week starting with germination. Images were taken per multiport tray with a 12 MP Panasonic DMC-FS10 digital camera (Kadoma, Japan) with ISO 100 and -2/3 exposure in a photo box that was placed over individual trays. Imaging stopped when 40% of plants from the control treatment had bolted. Additional images were taken before harvest. Images were analysed by an adapted script of Exposito-Alonso et al. (2018). From each image, two new images were produced, one retaining pixels in the range of green and the other in the range of red. The two images were then merged, the sum of pixels counted for each pot and time point, and the value transformed into mm^2^. For each plant, seven growth models were explored (linear, exponential, power, two- and three-parameter logistic, von Bertalanffy, and Gompertz) to fit the size data over time. Of these, the three-parameter logistic model–together with the more complex Gompertz model–was the best supported across plants and treatments. From the three-parameter logistic model we extracted the asymptote (maximum rosette size [mm^2^], *size*), the time to the inflection point (time to fastest growth [days], *x*_*mid*_), and the slope at the inflection point (*growth rate*). The script is accessible at github.com/heblackj/image_analysis.

Leaf and root functional traits. We stopped the experiment one month after 40% of the plants of the control group had started flowering. All plants were separated into four components, if present: inflorescences, dead leaves, living leaves, and roots. Leaves and roots were washed to remove attached soil and dried with a paper towel to remove excess water. The fresh weight of inflorescences, living leaves, and roots was taken. Then the material was dried separately for 48 h in an oven at 60 °C. We calculated the specific leaf area (*SLA*, size [mm^2^] per dry weight of leaves [mg], excluding dead leaves), the leaf dry matter content (*LDMC*, dry weight leaves [mg] per wet weight leaves [g], excluding dead leaves), and the root-to-shoot ratio (*root:shoot*; dry weight roots per dry weight all leaves and inflorescences). The range of trait values per treatment and family are presented in Online Resource 2 Table S3.

### Statistical analysis

To approach normality of the dependent variables, we log_10_-transformed growth rate, root:shoot ratio, SLA, and LDMC. An initial analysis of variance was performed to reveal the effects of days to germination, block, and tray within block on variables (Anova in car package; Fox and Weisberg 2019). If considerable variance was explained, variables were corrected for the specific effects. Furthermore, we looked into trait dependencies by correlating all traits within the central population at the level of the plant for each treatment separately (Fig. 3, rcorr in Hmisc; Harrell 2022) and performed a principal component analysis for each treatment (Online Resource 1 Fig. S2, factoextra package; Kassambara and Mundt 2020).

In the main analysis, we tested for the effect of temperature, watering and the interaction term on aspects of performance and functional traits using linear mixed effects models for continuous data or generalized linear models for binary data (lmerTest package; Kuznetsova et al. 2017). The random effects included population and family nested within population, but the precise structure was set based on model selection. The models that were compared by Akaike information criterion (AIC) varied from: including intercept, slope on temperature, slope on watering, and all covariances for population and family nested within population, to including intercepts only (results in Online Resource 2 Table S4). For each dependent variable the best model was chosen for final analysis. The random effects were evaluated by likelihood ratio testing (Table 1; lrtest in the lmtest package; Zeileis and Hothorn 2002). Differences in plant performance and traits between low- (SE, SW) and high-latitude populations (NE, NW, C) were tested by Wilcoxon rank sum tests (Table 2).

**Table 1:** Effect of heat and drought on performance and leaf and root functional traits of *Arabidopsis lyrata*

Variable	Estimates of fixed effects				Difference in log-likelihood					
	Intercept	Heat	Drought	Heat + Drought	Pop	Pop.*Heat	Pop.*Drought	Fam	Fam.*Heat	Fam.*Drought
Survival	3.51***	− 1.62***	− 1.84**	− 2.71***	0.81			2.96		
Flowering	− 0.33		− 1.76***		0.89			113.32***		
Size	1464.85***	− 209.25	− 374.06*	− 62.10**	< 0.01	1.85	2.51	< 0.01	43.02***	25.98***
x_mid_	27.12***	− 1.48***	− 1. 38***	− 2.30***	< 0.01	< 0.01	< 0.01	< 0.01	32.43***	14.89**
Growth rate	0.08***	0.01**	** < **− 0.01***	0.03***	< 0.01			109.62***		
SLA	1.04***	0.07	− 0.07	0.53***	< 0.01	1.87	1.9	< 0.01	43.64***	13.44**
LDMC	2.46***	− 0.06***	− 0.04***	0.04	< 0.01	< 0.01	< 0.01	< 0.01	12.70**	1.73
Root:shoot	0.13**	0.06*	0.05*	0.01	29.92**			54.64		

Estimates of fixed effects and the difference in log-likelihood for random effects are reported. Significance is indicated in bold (*P < 0.05, **P < 0.01, ***P < 0.001). Models for size, x_mid_ (time to fastest growth), SLA (specific leaf area) and LDMC (leaf dry matter content) assessed variances of intercepts, slopes on temperature and watering, and all covariances, for population and family (testing of an aspect included its variance and the two covariances). Models for survival, flowering, growth rate and root:shoot ratio assessed variances of intercepts only, for population (Pop.) and family (Fam.). The random-effects structure of models was determined based on model selection (Online Resource 2 Table S4)

**Table 2:** Effect of heat and drought on performance and leaf and root functional traits differing between southern and northern/central populations

Variable	P-values			
	Intercept	Heat	Drought	Heat + Drought
Survival	0.394	0.138	0.200	**0.004**
Flowering	0.721		0.964	
Size	0.252	0.268	0.483	0.661
x_mid_	0.781	0.417	0.806	0.621
Growth rate	0.515	0.760	0.081	0.495
SLA	**0.003**	0.064	0.133	**0.018**
LDMC	0.431	0.989	0.384	0.880
Root:shoot	**0.037**	** < 0.001**	** < 0.001**	0.312

x_mid_ is the time to fastest growth, SLA the specific leaf area, and LDMC the leaf dry matter content. P-values based on pairwise Wilcox tests are shown. Significant differences are indicated in bold (P < 0.05)

We conducted univariate and multivariate phenotypic selection analyses on the growth and functional traits of the central population with generalized linear models (de Jong 1995; Scheiner and Callahan 1999; Callaway et al. 2003). Trait data was standardized (mean = 0, deviation = 1) within treatment, and models were run for each treatment separately. An exception was the combined heat and drought treatment. As we lacked data on SLA, LDMC and root:shoot ratio of the many plants that had died in this treatment, we replaced values; we calculated family means for these traits under drought or heat treatment, averaged those values over the two treatments, and used this trait data instead in the selection analysis of the combined stress treatment. In models including single traits, we first evaluated the inclusion of both the linear and quadratic term by AIC (Table 3). As the inclusion of the quadratic term was rarely better, the multivariate models were built by only including linear terms (packages mcglm and htmcglm; Bonat 2018; de Freitas 2022). As fitn

**Table 3:** Selection analysis of plant growth, leaf and root functional traits under the four treatments, based on the performance measures [W] of flowering, survival or longevity

Variable	Univariate selection				Multivariate selection
	AIC_lin._	AIC_quad._	Coef._x_	Coef._x_^2^	Coef._x_
*Control; *W *= flowering [0/1]*					
Size	755	758	0.06**		− 0.02
x_mid_	761	761	0.02		− 0.02
Growth rate	761	761	− 0.01		− 0.03
SLA	290	309	0.33***		0.29***
LDMC	499	466	0.22***		0.10***
Root:shoot	624	567	− 0.19***		− 0.04*
*Heat;* W = *survival [0/1]*					
Size	553	552	< 0.01		< 0.01
x_mid_	546	547	0.03		< 0.01
Growth rate	549	549	− 0.03		< 0.01
SLA	− 27,626	− 27,621	< 0.01		< 0.01
LDMC	− 27,366	− 27,372	< 0.01	< 0.01	< 0.01
Root:shoot	− 27,385	− 27,394	< 0.01	< 0.01	< 0.01
*Drought;* W = *survival [0/1]*					
Size	623	623	0.02		< 0.01
x_mid_	613	621	0.06**		< 0.01
Growth rate	608	619	− 0.07***		< 0.01
SLA	− 24,473	− 24,476	< 0.01		< 0.01
LDMC	− 24,472	− 24,474	< 0.01		< 0.01
Root:shoot	− 24,805	− 24,806	< 0.01		< 0.01
*Heat* + *Drought;* W = *longevity*					
Size	1497	1512	0.18***		0.14**
x_mid_	1494	1502	0.16***		− 0.12
Growth rate	1448	1466	− 0.32***		− 3.6***
SLA_Heat&Drought_	1429	1429	− 0.07		− 0.11*
LDMC_Heat&Drought_	1432	1434	0.06		0.06
Root:shoot_Heat&Drought_	1449	1447	− 0.01		0.02

x_mid_ is the time to fastest growth, SLA the specific leaf area, and LDMC the leaf dry matter content. In the univariate selection models, each trait was explored for the importance of the linear and quadratic term by AIC, and for the model with the lower AIC, estimated coefficients are reported. The last column shows the estimated coefficients of a model of multivariate selection, with all six traits as linear effects. Significant coefficients (coef.) are indicated in bold (*P < 0.05, **P < 0.01, ***P < 0.001)

ess variables, we used the propensity to flower for the control treatment, survival for single stress treatments, and longevity for the combined stress treatment.

## Results

### Climate change

For the five populations studied, the climate had shifted between the periods of 1960–1990 and 2000–2018 (Online Resource 1 Fig. S1, Online Resource 2 Table S1). The change in mean temperature for the growing season of April to June and the summer months of June to August had increased by 0.4 °C and 0.6 °C, respectively. Change varied considerably among sites, e.g. for the summer means from + 0.1 °C to + 1.1 °C. At the same time, mean precipitation during April to June and June to August increased by 11 mm and 8.6 mm, respectively, again with some variability among sites. However, precipitation during the driest month of the year, which tends to be in late winter at the southern edge of *A. lyrata*, had declined by 14.5 mm. Under the conditions chosen in the experiment, we simulated average spring compared to summer temperature at the southern edge, and average spring precipitation compared to dry conditions, assuming that such extreme events may become more likely under global warming already during spring, when plants grow and start flowering.

### Heat and drought stress

**Fig. 2:**
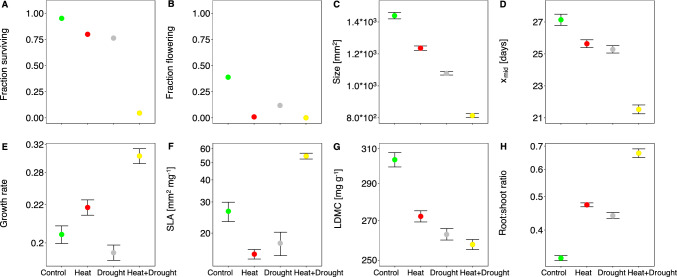
Effect of heat, drought and combined stress on performance and leaf and root functional traits of *Arabidopsis lyrata*. For each of the four treatment combinations of Control, Heat, Drought, and Heat + Drought, the overall corrected means with standard error (for non-binary traits) are shown. Please note the log_10_ scale for growth rate, SLA, LDMC, and root:shoot ratio

The treatments, temperature and watering, had strong additive and interaction effets (Table 1). Heat and drought lowered survival, and both stressors combined lowered survival even further (Fig. 2A). Longevity and the propensity to flower generally followed this pattern. The variable of longevity had low values and high variability in the treatment with combined stress (Online Resource 2 Table S3). For treatments with low temperatures, there was considerable flowering, and plants showed a lower propensity to flower under dry compared to control conditions (Fig. 2B).

Patterns for plant size were similar to those for survival. Maximum plant size was negatively affected by drought and – as a trend – by heat, and under combined heat and drought, their negative effect was exacerbated (Fig. 2C, Table 1). In turn, time to mid-size was shorter under single stress and interacted to be much shorter under combined stress (Fig. 2D). Furthermore, maximal growth rate was higher under heat and lower under drought, though the interaction term was again positive, indicating highly accelerated growth rates under combined heat and drought (Fig. 2E). LDMC decreased and the root:shoot ratio increased under single stress, indicating more water relative to dry weight in leaves and more relative investment into roots (Figs. 2G, H). However, the interaction term was not significant for the two traits. For SLA, only the interaction term was significant, indicating that plants had thinner leaves under combined heat and drought (Fig. 2F).

**Fig. 3:**
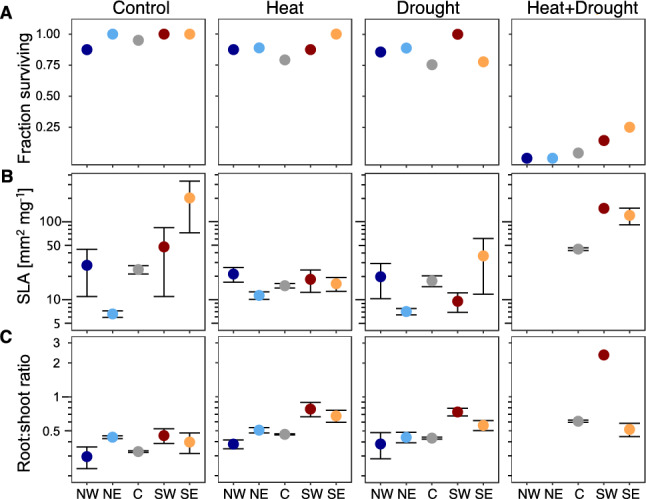
Effect of heat, drought and combined stress on performance and leaf and root functional traits of *Arabidopsis lyrata*. For each of the four treatment combinations, population corrected means with standard error are shown. The five populations are sorted on the x-axis from left/north to right/south. Please note the log_10_ scale for SLA and root:shoot ratio. SLA had a wider than usual range of values because leaf area was approximated by rosette surface area, resulting in particularly low values in the case of overlapping leaves and particularly high values in the case leaves had long petioles

Populations did not differ significantly in traits across treaments nor in response to drought or heat stress, except in the root:shoot ratio (Table 1). All other significant random effects involved families or how families reacted to heat and watering. Nevertheless, some trends of population differences could be detected based on contrasts between the southern and the more northerly populations, including the central population (Table 2). Survival was similar among populations across treatment combinations except for combined heat and drought; in that treatment, southern populations tended to perform better, indicating some adaptation to extreme heat combined with drought (Fig. 3A). Other traits that differed between the southern and all other populations were SLA and the root:shoot ratio. Plants of southern populations had higher SLA, particularly under combined heat and drought (Fig. 3B), as well as higher root:shoot ratios, and the ratio increased more under single stress (Fig. 3C).

**Fig. 4:**
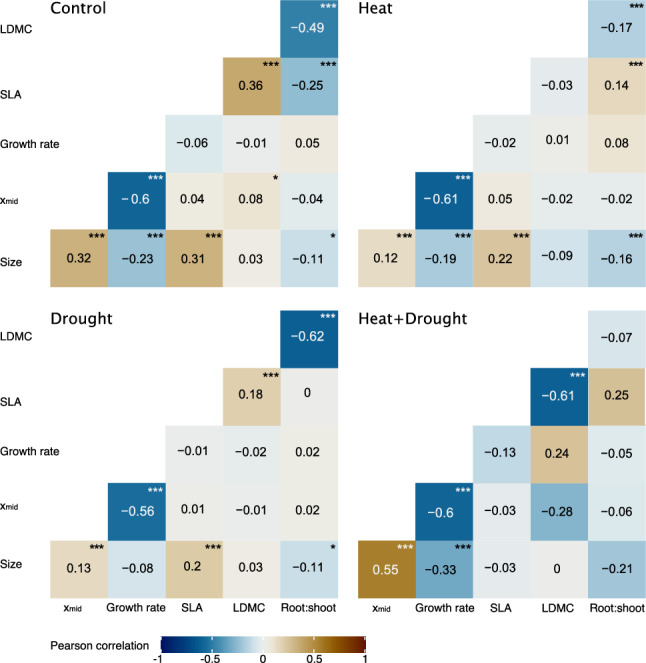
Phenotypic correlations between all trait pairs of the central population in the four treatments. Negative correlations are indicated in shades of blue, positive ones in brown. Colour intensity indicates the strength of the correlation. Significance is indicated (*P < 0.05, **P < 0.01, ***P < 0.001)

Correlations among traits were investigated for patterns within treatments by considering plants of the central population only (Figs. 4, Online Resource 1 S2). A few correlations were rather consistent across treatments, such as the negative correlation between maximal growth rate and both asymptotic plant size and time to mid-size, and the positive correlation between time to mid-size and plant size. There were two additional, consistently negative correlations both involving the root:shoot ratio, with plant size and LDMC.

### Traits under selection

Lastly, we investigated the traits under phenotypic selection under the different treatments (Table 3). Only the diverse central population was included in this analysis, as it covered most of the variation in traits of the edge populations. Under heat alone, no evidence for a trait under selection could be found, neither in the univariate nor in the multivariate selection analyses. Under drought, high x_mid_/late vegetative growth and a low growth rate were selectively favoured, though this was only found under univariate selection. Under combined heat and drought stress, we found evidence for positive linear selection favouring late maximal growth (univariate selection only), slow growth, large final size, and small SLA (multivariate selection only). Finally, under control conditions, we found evidence for positive linear selection favouring larger size (univariate selection only), higher SLA, higher LDMC, and lower root:shoot ratio.

## Discussion

Populations from the southern edge of the distribution of *A. lyrata* are affected by climate change, warmer average temperatures and more variable precipitation (Online Resource 2 Table S1). In our experimental study, we found that an increase in temperature and lower precipitation/watering had a negative effect on plant survival, and combined stress had a worse than additive effect on survival (Fig. 2A). Parallel findings were revealed for vegetative growth. Under single stress, plants had fast growth earlier and reached or tended to reach a smaller final size, while under combined stress, fastest growth happened even earlier and final size was smaller than if stressors had acted additively (Figs. 2C, D). Moreover, southern populations had a higher survival under combined stress compared to northern populations, indicating some adaptation to such extreme climatic conditions. We discuss these and further results below in regard to strategies for coping with climatic extremes and conflicts among strategies under variable climatic extremes at the low latitudinal edge.

Single stressors, heat or drought, lowered survival to a similar extent, though other aspects of performance differed. Size was reduced more under drought, but hardly any plants flowered under heat (Fig. 2B, C). The combination of heat and drought was then particularly devastating for plant survival, as stressors interacted in a synergistic manner. *Arabidopsis lyrata* must regularly experience very hot and dry conditions where it occurs. The species thrives in relatively open vegetation, on active sand dunes and on rocks with little vegetation cover, which heat up on sunny days. Furthermore, sandy soils typically have little water-holding capacity, and rocky outcrops have hardly any, except for cracks that may be filled with organic substrate. Given these features of the habitat, one would assume that the species can cope with both stressors, but apparently not when they co-occur as in our pot-design experiment. The result is in line with many studies showing that stressors multiply in their effect on plant performance (Mittler 2006; Zhang and Sonnewald 2017; Zandalinas and Mittler 2022).

We observed a number of plastic responses to heat, drought, and combined stress along the slow-fast continuum that did not seem adaptive. Plants exposed to heat or drought had the fastest growth early, a higher maximal growth under heat, and they reached a smaller final size (Figs. 2C–E, Table 1). This pattern of earlier and faster growth together with reduced size was strengthened under combined stress. Therefore, results suggest that *A. lyrata* generally responds to heat and/or drought by a strategy of escape in time (Levitt 1980; Ludlow and Muchow 1990) that seems to come at the cost of small size, in line with the concept of the slow-fast continuum (Reich 2014). The study of phenotypic selection indicated that these induced responses in vegetative growth were not adaptive or even maladaptive, with selection favouring opposite trait responses (Table 3). Under drought and combined heat and drought, selection tended to favour late and slow growth. Furthermore, under combined heat and drought, selection favoured large size. A reason could be that the plastic responses evolved in environments of short stress exposure, whereas the one applied in our study lasted longer and might have possibly favoured adaptations increasing climate tolerance (or resistance). Divergence between strategies of escape and tolerance have often been reported in response to drought stress. While early growth can be a drought escape or avoidance strategy with a short life cycle, plants with a tolerance strategy commonly grow more slowly under long-term drought stress and over a longer period of time, and thus live longer (Franks 2011; Tardieu 2012; Bouzid et al. 2019; Csilléry et al. 2020; Burnette and Eckhart 2021).

Small size need not necessarily be a cost of early and rapid growth but could be beneficial under heat and drought. Under heat, small leaves rather than large ones are more likely to maintain a low leaf temperature by higher transpiration (Vile et al. 2012; Stewart et al. 2016; Saini et al. 2022). Under drought, small leaf size can be beneficial as water loss is lower (Lin et al. 2017). Such benefits may have also partially existed in our experiment, as under heat or drought alone we found no sign of positive selection for larger size (Table 3). Moreover, small size seems largely a cost of early and fast growth. Phenotypic correlation analysis on the central population supported that the three traits of time to fastest growth, maximal growth rate and final plant size, were strongly integrated in each of the four treatment combinations used in our study, with the strongest found under combined stress (Fig. 4). Therefore, while small size may be of some advantage under single stress, it is a serious cost to early and rapid growth under combined stress.

We also observed plastic responses in leaf and root functional traits. Plants had a higher root:shoot ratio and more water in leaves (lower LDMC) under single stress and thinner leaves (higher SLA) under combined stress (Table 1). Morphological adaptations to maintain a high water potential under stress are typically achieved by increased root systems, reduced vegetative growth or reduced stomatal transpiration loss, e.g. by thicker leaves (Sicher et al. 2012; Maggio et al. 2018; Seleiman et al. 2021). Alternatively, tolerance strategies are associated with maintaining hydrostatic pressure, by osmotic adjustments, and cavitation resistance (Delzon 2015; Blum 2017). Except for thinner leaves being disfavoured under combined heat and drought (in multivariate selection analysis only), none of the three leaf and root functional traits were found to be under selection under single or combined stress while they were under control conditions. Under control conditions, a high root:shoot ratio was negatively selected against, indicating costs. Furthermore, thin leaves (higher SLA) with a high dry matter content (higher LDMC) – potentially photosynthetically highly active – were favoured. Plants seem to adjust plastically in response to stress mainly by trait expression away from what is favoured under benign conditions.

However, southern populations, which had the highest survival under combined heat and drought, differed exactly in leaf and root functional traits. The two northern populations had no survival under combined stress, the central population, represented by many more plants in the experiment, had some survival, and the two southern-range-edge populations had considerable survival (Fig. 3A, Table 2). The southern populations seem to have been pre-exposed to similar stress conditions in the past and adapted to them. Therefore, traits that we found divergent between southern and more northern populations can indicate the traits of adaptation (Estarague et al. 2022). Southern populations differed in the expression of a higher root:shoot ratio, especially under stress (Fig. 3C). This response of low-latitude populations in the root system should allow the cooling by transpiration while maintaining the leaf water potential and photosynthesis (Stewart et al. 2016; Berny Mier y Teran et al. 2019; Csilléry et al. 2020; Marchin et al. 2022). Furthermore, under combined heat and drought, plants mainly from a southern population had thinner leaves (higher SLA, Fig. 3B, Table 2). This latter finding is hardly an adaptation, however, as thicker leaves were shown to be better at heat buffering and low water loss by evaporation (Wright et al. 2005; Leigh et al. 2012; Zhou et al. 2020), leaving the root:shoot ratio as the most likely candidate.

In fact, the combination of results of the different analyses suggests some important differences in the root:shoot ratio between southern and northern populations. At a first glance, the presumably adaptive differences between the southern and northern populations are in line with induced responses by stress – higher root:shoot ratio under single stress and higher SLA under combined stress (Table 1), but with selection not found to act on these traits (Table 3). However, a high root:shoot ratio can be achieved by either investing less in shoots or investing more in roots. The plastic response of an increased root:shoot ratio under single stress may have been the result of smaller plant size and lower investment in shoots, which was neither disfavoured nor favoured by selection in those environments. In line with this, thin leaves, as found under combined heat and drought, may indicate less investment in above-ground structures as compared to roots (Wright et al. 2005; de Castro et al. 2019), which was not an adaptation but actually disfavoured in that environment (under negative selection in multivariate selection analysis). It is important to emphasize that these results were found with a focus on the central population. Southern populations are probably different in that they had a high root:shoot ratio owing to a higher investment in root structures and that is why they performed better under stress. Evidence in favour of this is their higher root:shoot ratio, particularly under stress, that is not paralleled with a lower investment in above-ground plant size (Table 2). The results clearly indicate the need to study the evolutionary potential of root traits in the context of southern range limits and climate change (Zhou et al. 2019; Taseski et al. 2021).

## Conclusion

We studied replicate *A. lyrata* populations from across its distribution for their ability to cope with single stress, heat or drought as well as combined heat and drought as can be expected at the southern range edge under global warming. Our results led to two main conclusions for the species. First, the combination of heat and drought reduces plant survival more than predicted by the additive effects of heat and drought. Second, while plants from the north cannot persist under such conditions, plants originating from the southern end of the range have some survival, indicating the potential for adaptation. Selection analysis with a focus on the central population suggested that plastic responses to heat and drought followed a strategy of escape, which was not favoured under any of the stress environments. In line with this, the higher stress tolerance of the southern populations did not involve adjustments on the slow-fast continuum but was probably achieved by a higher allocation into roots as compared to shoots.

### Supplementary Information

Below is the link to the electronic supplementary material.

Supplementary file1 (XLSX 20 KB)

Supplementary file2 (PDF 1396 KB)

## Data Availability

The data will we available at figshare (10.6084/m9.figshare.23104481).

## Code availability

The code will be available on figshare (10.6084/m9.figshare.23104481).
