# Supplementary material for: Negative interaction effect of heat and drought stress at the warm end of species distribution
Source: Oecologia. 2024 Jan 23;204(1):173–85. doi: 10.1007/s00442-023-05497-5 (PMC10830594; doi:10.1007/s00442-023-05497-5)
Supplement: Supplementary file 2 — Supplementary file2 (PDF 1396 KB) [file 442_2023_5497_MOESM2_ESM.pdf]

Digital supplementary material 1

Journal: Oecologia

Tittle: Negative interaction effect of heat and drought stress at the warm end of species distribution

Authors: Judith R. Schepers*, Jessica Heblack, and Yvonne Willi

*Correspondence: Judith.schepers@unibas.ch

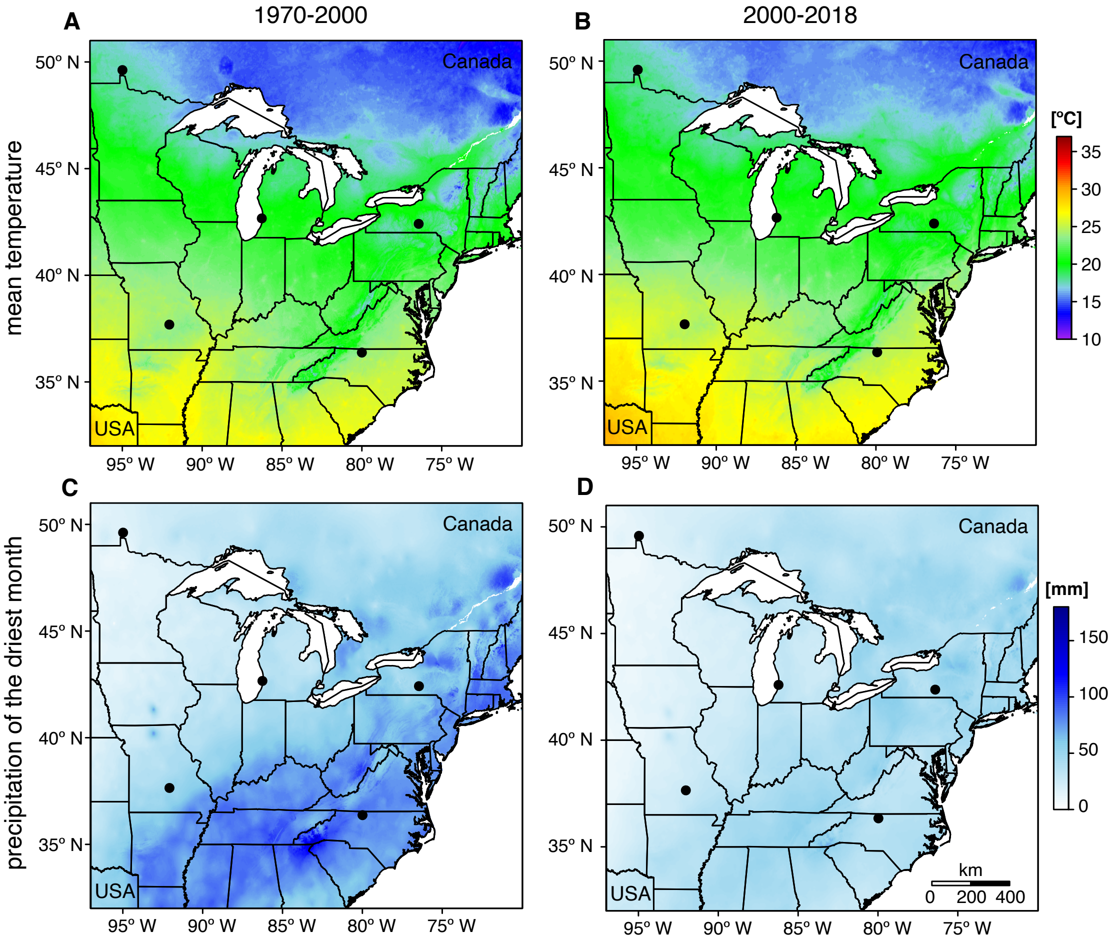

Fig. S1: Maps representing climate of different periods of the recent past in the area of distribution of North American *Arabidopsis lyrata*. A) Mean temperature averaged for April, May and June in the time period of 1970-2000 and B) mean temperature averaged for June, July and August in the time period of 2000-2018 in ºC (scale of colours on the right). C) Precipitation during the driest month in the time period of 1970-2000 and D) of 2000-2018 in mm. Black dots indicate the location of populations used in this experiment. The border between the US and Canada, as well as US state borders are indicated by black lines.

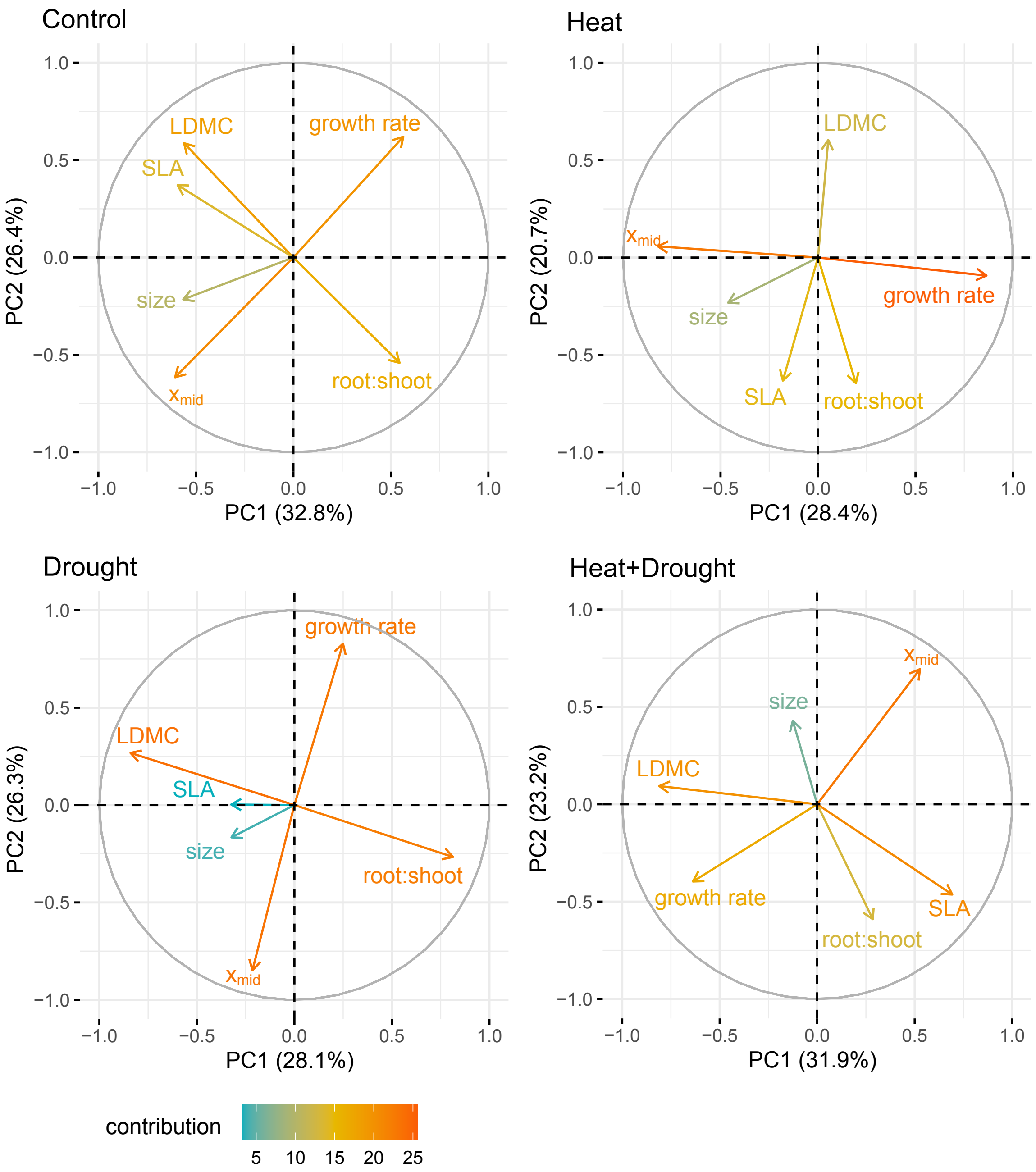

Fig. S2: Principial component analysis on plant growth, leaf and root traits of a diverse *Arabidopsis lyrata* centre population for four temperature and watering treatments - Control, Heat, Drought, and Heat+Drought. Arrow length and color represent the contribution of each trait to the first two principal component axes (with the amount of phenotypic variation they depict written in parenthesis).
